# Effects of Plant-Based Extract Mixture on Alcohol Metabolism and Hangover Improvement in Humans: A Randomized, Double-Blind, Paralleled, Placebo-Controlled Clinical Trial

**DOI:** 10.3390/jcm12165244

**Published:** 2023-08-11

**Authors:** Seung Hee Jung, Yun Ha Lee, Eun Kyu Lee, Soo-Dong Park, Jae-Jung Shim, Jung-Lyoul Lee, Hye Hyun Yoo

**Affiliations:** 1 R&BD Center, hy Co., Ltd., 22, Giheungdanji-ro 24beon-gil, Giheung-gu, Yongin-si 17086, Republic of Korea; jungsh@hy.co.kr (S.H.J.); yunha1006@hy.co.kr (Y.H.L.); soodpark@hy.co.kr (S.-D.P.); jjshim@hy.co.kr (J.-J.S.); 2 Institute of Pharmaceutical Science and Technology, College of Pharmacy, Hanyang University, 55, Hanyangdaehak-ro, Sangnok-gu, Ansan 15588, Republic of Korea; giddyg@naver.com

**Keywords:** plant-based extract mixture, hangover, Acute Hangover Scale, alcohol metabolism, alcohol dehydrogenase, acetaldehyde dehydrogenase

## Abstract

Hangovers are uncomfortable physiological symptoms after alcohol consumption caused by acetaldehyde, a toxic substance in which alcohol is metabolized by alcohol dehydrogenase (ADH). Rapid alcohol and acetaldehyde decomposition are essential to alleviate alcohol handling symptoms. This study investigated the effects of HY_IPA combined with *Mesembryanthemum crystallinum, Pueraria lobata flower*, and *Artemisia indica* on alleviating hangovers. A randomized, double-blind, parallel-group, placebo-controlled clinical study was conducted on 80 individuals with hangover symptoms. Alcohol intake was 0.9 g/bw with 40% whiskey, adjusted proportionately to body weight. The Acute Hangover Scale total score was 5.24 ± 5.78 and 18.54 ± 18.50 in the HY_ IPA and placebo groups, respectively (*p* < 0.0001). All nine indicators of the hangover symptom questionnaire were significantly improved in the HY_IPA group (*p* < 0.01). Blood alcohol and acetaldehyde concentrations rapidly decreased from 30 min in the HY_IPA group (*p* < 0.05). ADH and acetaldehyde dehydrogenase (ALDH) activities in the blood of the HY_IPA group were significantly higher than those in the placebo group at 0, 1, and 2 h after alcohol consumption (*p* < 0.01). The rapid hangover relief was due to increased ADH and ALDH. Therefore, HY_IPA effectively relieves hangover symptoms by decomposing alcohol and acetaldehyde when consumed before alcohol consumption.

## 1. Introduction

Hangover is defined as “the combination of mental and physical symptoms, experienced the day after a single episode of heavy drinking, starting when blood alcohol concentration approaches zero” [1]. Alcohol has various effects on liver metabolism depending on the amount consumed. When alcohol is absorbed in the stomach and small intestine and enters the liver through the blood, it is metabolized to acetaldehyde by alcohol dehydrogenase (ADH). Acetaldehyde is oxidized by acetaldehyde dehydrogenase (ALDH) to acetic acid and excreted as urine and CO_2_ [2,3,4]. Intermediate metabolites such as acetaldehyde and nicotinamide adenine dinucleotide, generated during alcohol oxidation, cause various physiological changes, leading to metabolic and alcoholic liver diseases [5]. Hangovers are the side effects of acetaldehyde that are not metabolized by the liver. The physical symptoms of hangovers include thirst, sleepiness, headache, fatigue, and sweating, whereas the psychological symptoms include dizziness, depression, anxiety, and irritability [6].

As alcohol consumption increases in modern society, the interest in beverages and functional ingredients for hangover relief is increasing. The market for liver protection and hangover relief has increased recently, and many studies have explored possible treatments or substances that could alleviate hangover symptoms [7]. However, there is no pharmacological agents of hangover on the market with clear evidence of efficacy. Since ancient times, aspirin and paracetamol have been used to relieve minor headaches and pains during hangover [8], but these common analgesics have not been evaluated in clinical trials for hangover [9]. According to some studies, it is known that the higher the frequency of hangovers, the more likely they are to be diagnosed with alcohol use disorder later [10]. Multifarious drugs are available to treat alcohol use abuse, such as disulfiram (aldehyde dehydrogenase inhibitor), naltrexone (opioid antagonist), topiramate (GABAergic anticonvulsant), and acamprosate (N-methyl-d-aspartate/glutamate receptor modulator) [11,12,13]. In addition, these drugs allow you to voluntarily reduce your alcohol intake and cravings. However, they have severe side effects compared to natural products, such as ataxia, impaired attention, and poor consciousness. A lot of studies on the efficacy of natural materials for alcohol hangover relief have been conducted. Plant-derived compounds significantly reduce alcohol intake, cravings, and withdrawal symptoms [14].

For instance, *Mesembryanthemum crystallinum* (ice plant) has antioxidant effects, radical scavenging, nitric oxide inhibitory, hyperglycemic, and memory-improving activities [15]. In addition, ice plants produce gamma-aminobutyric acid (GABA) via lactic acid fermentation and are used as functional food ingredients [16]. GABA has various physiological activities, such as suppressing the increase in cholesterol and triglyceride levels in the blood, lowering blood sugar, and promoting alcohol metabolism [17]. *Pueraria lobata flower* are dried kudzu flowers widely used in folk medicine to treat thirst, headache, vomiting, and mental confusion after drinking [18]. *Pueraria lobata flower* can alleviate hangover symptoms and has long been used to treat chronic alcoholic liver damage in traditional Chinese medicine. In addition, these flowers have been used to treat disorders such as alcohol abuse [19]. *Artemisia* herbs are used as food additives and traditional medicines to treat various liver related diseases [20], for example liver cancer, and cirrhosis [21]. The antioxidant capacity of herbal ingredients can be used as an effective treatment to suppress hangover symptoms and reduce alcohol absorption [22].

Many studies have explored possible treatments or substances to alleviate hangover symptoms [23]. In particular, the liver function improvement and alcohol decomposition effects of natural products have been studied through in vivo or in vitro studies, but their hangover-relieving efficacy has not been reported in detail in clinical trials. The aim of this study is to provide an overview of effective natural materials for the prevention and treatment of severe physical symptoms of hangover caused by alcohol consumption. Thus, in this study, a clinical trial was carried out in healthy adults to compare and evaluate the efficacy of HY_IPA in improving symptoms and relieving hangovers caused by alcohol intake.

## 2. Materials and Methods

### 2.1. Study Participants and Inclusion/Exclusion Criteria

This clinical study participants were recruited from H Plus Yangji Hospital (Seoul, Republic of Korea). The inclusion criteria were as follows: (1) aged 19–40 years old, (2) body mass index (BMI) of 18.5–25 kg/m^2^, (3) hangover experience after drinking in the past month, (4) result of exhaled alcohol test on 0.00% factor at the morning visit 2 (day 0), and (5) consent to participate in the human clinical trial and contacting the person who signed the informed consent.

Patients were excluded if they were treated for cardiovascular (high blood pressure), immune, respiratory, endocrine (diabetes), gastrointestinal, liver, biliary tract, kidney and urinary, nervous, and musculoskeletal system, psychiatric, infectious, and thyroid diseases, malignant tumors, or a history of peptic ulcer, reflux esophagitis, or gastrointestinal surgery. 

Patients were not eligible for participation in this study if they were pregnant or planning to become pregnant, were nursing mothers, had an alcohol use disorder, alcoholism, a positive factor of substance abuse qualitative test, or were taking an anti-abuse drug or warfarin, clopidogrel, aspirin, or non-steroidal anti-inflammatory drugs. Participants were excluded if they had abnormal creatinine (two times the standard upper limit), aspartate aminotransferase (AST), or alanine transaminase (ALT) (three times the standard upper limit) levels. Patients who participated in other interventional clinical trials within 1 month or planned to participate in other interventional clinical trials, were sensitive or allergic to the ingredients of this clinical trial, or persons deemed inappropriate by the investigator for any other reason were also excluded.

### 2.2. Study Design

This study was performed from 5 October to 5 November 2022 after receiving approval from the Clinical Trial Review Board of the H Plus Yangji Hospital (IRB file no. HYJ2022-08-025) and registered with the Clinical Research Information Service (CRIS, no. KCT0008402) of the Korea Disease Control and Prevention Agency, and supported by the Ministry. This clinical trial was designed as a randomized, double-blind, paralleled, placebo-controlled trial. The placebo and plant-based extract mixture groups (HY_IPA) were randomly assessed on the day of the selection evaluation and administered according to a double-blind procedure. The randomization lists were created by a statistician using computers with the sponsor. Participants and investigators were blinded to the intervention assignments until the end of the study. The priori power analysis was performed to estimate the sample size required by the superiority test. In previous studies, the mean and standard deviation of the AHS score in the treatment group (mean 0.8, standard deviation 0.3) and placebo group (mean 1.5, standard deviation 0.9) [24]. The allocation ratio of each group 1:1, at alpha = 0.05, the power of 80%, and the minimum of 30 participants in each test group was calculated as the suitable number of participants for clinical research. The total number of participants required to establish effectiveness was estimated to be 80, considering the dropout rate (25%). Participants were sent for a human application test on visits 1 and 2 (Day 0). A demographic, lifestyle, medical history, and drug administration history survey, physical examination, vital signs (blood pressure and pulse), physical measurements (height, BMI, weight), clinical pathology test, pregnancy response test (only for women of childbearing age), and drinking habit questionnaire including questions such as type of alcohol, amount consumed, how many times per week, and experience of hangover in the past month were combined to evaluate and record whether the participant was suitable for the selection/exclusion criteria. Assigned as a test or control group, clinical trial participants were single-dosed with the test or control food at visit 2 (Day 0). The allocation ratio of each group was test group: control group = 1:1.

### 2.3. Interventions

All the test materials were provided by hy Co., Ltd. (Yongin, Republic of Korea). We used a beverage (each 80 mL/bottle) containing 240 mg malt extract (natural brown pigment) placebo or 700 mg plant-based extract mixture (*M. crystallinum*, *P. lobata flower*, and *A. indica*) dissolved in water for the control and intervention groups, respectively. Other ingredients were included equally (honey jujube flavor 80 mg, and stevia 24 mg). Each sample was identical in appearance, shape, color, flavor, sweetness, packaging, and additive. All participants were provided the same meal before the trial and consumed the placebo or intervention beverage at 2 h after meal ingestion. Thirty minutes after ingestion of the test substance, whiskey with 40% alcohol content was consumed (alcohol content 0.9 g/bw), and shrimp snacks (20 ea) within 30 min. Participants stayed overnight in the clinical research center and all participants slept 6 h after alcohol intake. Study staff were required to monitor the participant’s safety throughout the night. The participants slept in bed for 6 h with the lights off. After waking up next morning, they filled out a hangover symptom questionnaire at 12 h after alcohol intake.

### 2.4. Outcome Measures

#### 2.4.1. Primary Outcome Measure: Acute Hangover Scale 

A survey of hangover symptoms and an assessment of hangover severity was performed. The Acute Hangover Scale (AHS) consists of 9 items covering the severity of hangover symptoms and overall hangover severity rating, all of which are rated from 0 to 7, with an AHS total score calculated as the average of all items. The anchors were ‘none’ (score = 0), ‘mild’ (score = 1), ‘moderate’ (score = 4), and ‘incapacitating’ (score = 7). 

#### 2.4.2. Secondary Outcome Measure: Alcohol and Acetaldehyde Analysis in the Blood 

##### Sample Handling and Collection

Blood was collected at 0, 0.25, 0.5, 1, 2, 4, 6, and 13 h after alcohol intake. A catheter filled with saline for injection was installed in the vein area of the participant’s arm, and 5 mL of blood was collected at each point. The blood was placed in a BD Vacutainer^®^ NaF tube and used for analysis immediately while maintaining the temperature at 4 °C.

##### Alcohol and Acetaldehyde Analysis in Blood

All clinical blood samples were stored on ice to analyze ethanol and acetaldehyde. For clinical blood analysis, 200 μL of human plasma was added to 500 μL of saturated NaCl solution in the headspace vial, and 100 μL of n-butanol (0.005%) was added. Human blood samples were measured using Agilent 5977 series GC systems (Agilent Technologies, Palo Alto, CA, USA) equipped with a CTC headspace GC/MS detector (CTC analytics AG, Zwingen, Switzerland). Chromatographic separation of ethanol, acetaldehyde, and n-butanol was achieved using Discovery HP-INNOWAX columns (0.32 mm × 30 m, 0.5 μm, Sigma-Aldrich, St. Louis, MO, USA). Helium was used as the carrier gas and maintained at a constant flow rate of 3 mL/min, while the interface temperature was set at 200 °C. For GC-MS detection, an electron ionization system with an ionization energy of 70 eV was employed. The GC oven temperature was initially held at 35 °C for 3 min, ramped up to 85 °C at a rate of 40 °C per minute, and then held for 2 min. The equilibrium temperature and time in the headspace sampler were 70 °C and 10 min, respectively. The injection volume was 250 μL and the split ratio was 100:1. The mass spectrometer operated in single ion monitoring mode, with ethanol set at m/z 45, 46, and 31, and acetaldehyde set at m/z 43, 41, and 29. The quantification of ethanol and acetaldehyde was based on m/z 45 and 43, respectively.

### 2.5. Safety

Vital signs such as blood pressure (systolic and diastolic blood pressure), body temperature, pulse rate, and clinical laboratory tests (hematology, biochemical, and urine tests) were performed on the participants at screening (visit 1) and visit 2 (day 1). In addition, during the study period, adverse events were confirmed through interviews or questionnaires with the participants.

### 2.6. ADH and ALDH Enzyme Activity Analysis 

Alcohol hangover markers such as ADH and ALDH activity were measured after separating the blood into plasma. Each commercial kits were used to determine ADH (K787; Biovision, Milpitas, CA, USA) and ALDH activity (K731; Biovision, Milpitas, CA, USA) in the plasma. All analytical procedures were carried out in accordance with the manufacturer’s instructions. 

### 2.7. Statistical Analysis 

Statistical analyses were performed using SAS version 9.4, SAS Institute, Cary, NC, USA. The data obtained in this clinical trial were determined by calculating the means ± standard deviation (SD) with appropriate descriptive statistics, and the significance of the difference was two-tailed at *p* < 0.05. A normality test (Shapiro–Wilk test) was conducted to compare groups, and a two-sample *t*-test was performed when normality was satisfied in the HY_IPA and placebo groups. A comparative analysis was performed using the Wilcoxon rank-sum test when one group was not normally distributed. A paired *t*-test was used for comparisons within groups. Categorical variables were compared using the chi-squared test or Fisher’s exact test. For correlations with 1-item hangover score and usual alcohol consumption secondary outcomes, Spearman’s correlations were computed.

## 3. Results

### 3.1. Enrollment

**Figure 1:**
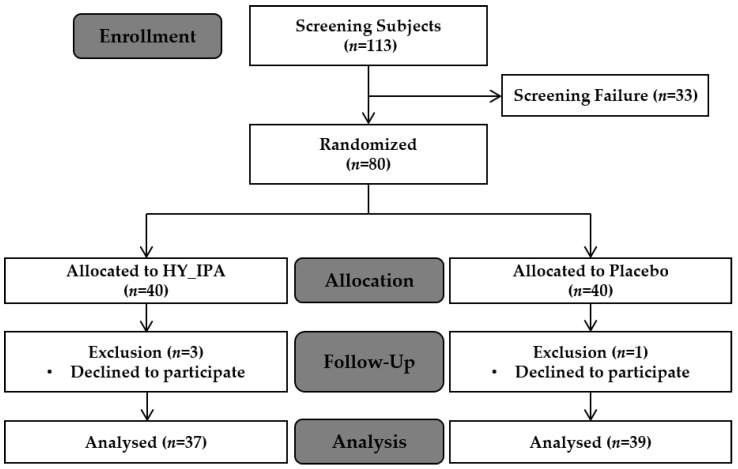
Flow diagram showing the selection and allocation of participants in the study.

A total of 80 participants were enrolled after excluding 33 people during screening, and 76 participants were included in the experimental analysis during the period 5 October 2022 to 5 November 2022. During the trial, four participants (three in the HY_IPA group and one in the placebo group) were excluded from the final analysis (Figure 1). Of these, three participants in the HY_IPA group and one in the placebo group withdrew consent.

No participants were excluded from the per-protocol (PP) analysis set among the participants of the full analysis (FA) set; therefore, both FA and PP sets were analyzed with the same number of cases. The PP set was the primary analysis subject to evaluate the efficacy, and 76 participants (37 in the test and 39 in the control group) were included in the clinical trial.

### 3.2. General Participant Characteristics 

Table 1 compares the demographic information and pre-intake characteristics of the participants. All characteristics before intake, including the demographic information of the participants, were compared between the intake groups to identify the differing factors [25].

A total of 23 males (62.16%) and 14 females (37.84%) comprised the test group. The control group included 22 males (56.41%) and 17 females (43.59%); however, there was no significant difference between the intake groups. The test group averaged 29.00 ± 5.70 years, and the control group averaged 28.69 ± 4.59 years, showing no statistically significant difference in age between the intake groups.

In addition, no statistically significant differences between the intake groups regarding childbearing status (female only), exercise status, quantity, and frequency of alcohol consumption, smoking status, height, or BMI were observed; thus, comparability between groups can be assumed.

### 3.3. Biochemical Parameters

Safety evaluation was performed with safety set analysis as the primary analysis; 37 people in the test group and 39 in the control group were randomly assigned to the human application test. No statistically significant difference in the hematological examination items was observed. As a result of ALT analysis during the blood chemistry examination, the HY_IPA increased by 1.73 ± 3.65 U/L (*p* = 0.0066), and the control group increased by 0.28 ± 6.75 U/L (*p* = 0.7956), showing a statistically significant difference between intake groups (*p* = 0.0323) (Table 2). However, this change was within the normal range, and no clinical significance was confirmed. In addition, no statistically significant differences after one day of food intake were observed regarding vital signs and body measurements (Table 2). Furthermore, no significant difference in adverse reactions between the intake groups was observed, and no severe adverse reactions occurred. Therefore, no problem with safety was reported.

### 3.4. Survey of Hangover Symptoms 

The AHS scores after alcohol consumption were compared between the test and control groups to evaluate whether there was a statistically significant difference. Table 3 presents AHS results measured 12 h after alcohol intake. The AHS score was 5.24 ± 5.78 in the HY_IPA group and 18.54 ± 18.50 in the control group, showing a significant difference between the intake groups (*p* < 0.0001 [W]). Compared with the placebo group, the HY_IPA group showed significantly reduced scores in ‘hangover’, ‘thirst’, ‘tired’, ‘headache’, ‘dizziness/faintness’, ‘loss of appetite’, ‘stomachache’, ‘nausea’, and ‘heart racing’. Additionally, we confirmed differences in hangovers and sex (Table 4). When comparing the male and female groups on the AHS, both groups showed significant differences in the total score (*p* < 0.05). The indicators that significantly improved in males and females were ‘hangover’, ‘thirst’, and ‘tired’ (*p* < 0.05). In the male group, except for indicators showing significant differences in common with the female group, symptoms improved in ‘dizziness/faintness’, ‘loss of appetite’, ‘stomachache’, and ‘heart racing’ (*p* < 0.05). Additionally, in the female group, symptoms improved regarding ‘nausea’ (*p* < 0.05).

### 3.5. Change in Blood Alcohol and Acetaldehyde Levels

**Figure 2:**
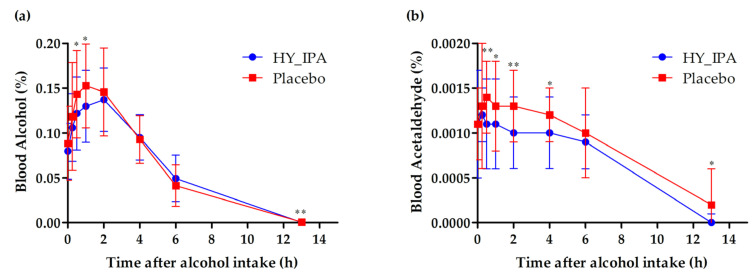
Effects of HY_IPA and the placebo on the alcohol and acetaldehyde concentration in the blood after alcohol consumption: (**a**) alcohol levels in the blood; (**b**) acetaldehyde levels in the blood. Compared between groups; *p*-value for two-sample *t*-test (T) or Wilcoxon rank sum test (W), * *p* < 0.05, ** *p* < 0.01.

Figure 2 shows the results of measuring the changes in blood alcohol and acetaldehyde concentrations from 0 to 13 h after drinking. 

The peak alcohol blood concentration (C_max_), time to reach the peak blood concentration (T_max_), and area under the curve (AUC) from 0 to 13 h after drinking are shown in Table 4. The blood alcohol concentration was significantly lower in the HY_IPA group (*p* < 0.05) compared to the control group at 0.5 and 1 h (Figure 2a). The T_max_ (*p* < 0.05) and the C_max_ also tended to be decreased in the HY_IPA group (*p* = 0.0586). Blood acetaldehyde levels were significantly lower in the HY_IPA group (*p* < 0.05) than in the control group at 0.5, 1, 2, and 4 h (Figure 2b), and there was a significant decrease (*p* < 0.05) in the AUC and C_max_ (Table 5).

### 3.6. ADH and ALDH Activity in the Blood 

**Figure 3:**
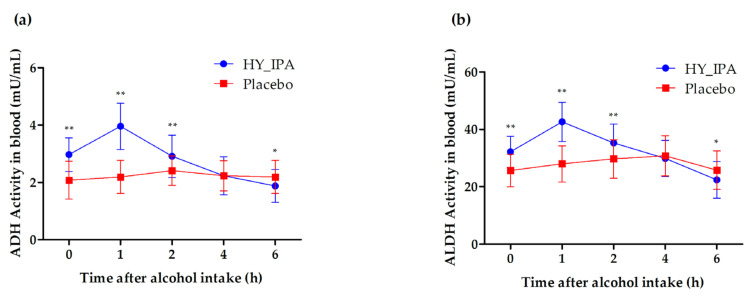
Effects of HY_IPA and the placebo on the ADH and ALDH concentration in the blood after alcohol consumption: (**a**) alcohol levels in the blood; (**b**) acetaldehyde levels in the blood. Values are expressed as means ± SEM. *p*-values are from nonparametric paired *t*-tests. * *p* < 0.05, ** *p* < 0.01.

Figure 3 shows the changes in blood ADH and ALDH concentrations over time after alcohol intake for 6 h after drinking. There was a significant difference between the control and HY_IPA groups at 0, 1, 2, and 6 h, but not at 4 h after alcohol consumption. ADH (Figure 3a) and ALDH (Figure 3b) activities in the blood were significantly higher in the HY_IPA group than in the placebo group at 0, 1, and 2 h (*p* < 0.01) and 6 h (*p* < 0.05) after alcohol intake (Table 6 and Table 7).

### 3.7. The Association of Hangover Score and Secondary Outcomes after Alcohol Consumption

When analyzing the data for the HY_IPA group, a clear distinction became evident. The 1-item hangover severity score, a significantly correlate with concentration of alcohol, acetaldehyde, and ADH, ALDH activity at 1 h after alcohol consumption. Alcohol (r = 0.3570, *p* = 0.0301), acetaldehyde (r = 0.4335, *p* = 0.0074), ADH (r = −0.1529, *p* = 0.3662), and ALDH (r = 0.489, *p* = 0.0021). When analyzing the data for the HY_IPA group, a clear distinction became evident (Table 8). 

## 4. Discussion

In this study, clinical trials with hangover-relieving drinks made from the extracts of medicinal plants (*Mesembryanthemum crystallinum*, *Pueraria flower, Artemisia indica*) were conducted to confirm the efficacy of alcohol decomposition in the human body and to relieve hangover symptoms. Blood alcohol concentration can identify changes in the human body due to alcohol consumption. However, hangover symptoms caused by alcohol consumption intake begin when the blood alcohol concentration decreases and persists after the blood alcohol disappears. Therefore, research is being conducted using a questionnaire to evaluate hangover symptoms [26]. Our survey included nine indicators, and significant improvement effects was observed in all nine indicators (*p* < 0.05), confirming that the symptoms were significantly improved in the HY_IPA group. 

Most hangover studies have not investigated sex differences separately. Until 2004, studies on the pathology and physiological correlation of alcohol-induced hangovers were studied with mainly male participants [27,28,29,30,31,32,33]. Thus, our study confirmed hangovers and sex differences. A comparison of the male and female groups on the AHS showed significant differences in total scores. In addition, in females, symptoms improved in the nausea group. In another study, female reported higher severity scores for fatigue, weakness, dizziness, and nausea than male [34]. In previous studies, the Hangover Symptoms Scale (HSS) questionnaire was used to assess hangover symptoms, and female had higher HSS total scores. These results indicate that women may be more vulnerable to severe hangover symptoms than men [35]. In addition, these results have pharmacological implications, since women weigh less and have a lower body water percentage than men. Therefore, they can achieve higher intoxication degrees and, presumably, more hangovers per unit of alcohol [36,37]. Therefore, hangover relief efficacy can show substantial differences between the sexes, and a test with as many males and females in the same ratio as possible should be conducted in a hangover clinical trial. In this study, we tried to ensure that the ratio of men and women was similarly constructed, and as a result, it was confirmed that hangover symptoms significantly improved more in the HY_IPA group than in the placebo group in all items of the AHS questionnaire. These results confirmed that HY_IPA was effective in relieving hangover symptoms. The original studies claimed that alcohol can cross the blood–brain barrier (BBB), but not the case of acetaldehyde [38]. According to previous research suggests that the amount of ethanol (not acetaldehyde) present in the blood is an important determinant of hangover severity [39,40]. However recent research suggests that systemic administration of alcohol and acetaldehyde can increase the accumulation of acetaldehyde in the blood and brain of mice [41]. A recent clinical trial that reported a correlation between acetaldehyde concentration and hangover symptom [42,43,44,45,46,47]. These were used a highly sensitive GC-MS method that reliably measures acetaldehyde concentrations [48]. This may be because blood levels of acetaldehyde often fall below detection limits during a hangover. Additionally, genetic variations, such as the diversity of acetaldehyde detoxifying alleles, influences the severity of hangovers. For example, in populations of Asian descent with ALDH2*2, the alleles may cause the experience of worse hangovers [49].

Acetaldehyde, a primary product of alcohol metabolism, is not oxidized to acetate and remains in large amounts in the body, thus, causing hangover symptoms. Therefore, we measured blood alcohol and acetaldehyde concentrations as a secondary validity of this clinical study to confirm the hangover improvement effect of HY_IPA. The blood alcohol concentration was significantly decreased in the HY_IPA group compared to the placebo group. Furthermore, the statistically significant difference in T_max_ and the C_max_ tended to be lower in the HY_IPA group. The content of acetaldehyde in blood was significantly decreased in the HY_IPA group than in the control group, and a significant decrease in the AUC and C_max_ was observed. These results indicate that HY_IPA effectively reduces the concentration of alcohol absorbed into the body and the concentration of acetaldehyde generated during alcohol metabolism, confirming that HY_IPA can alleviate hangover symptoms after alcohol consumption. 

Excessive acetaldehyde in the blood can move to other organs, including the brain, and has harmful effects. Therefore, to decompose ingested alcohol quickly or expel it from the body is essential [50,51]. Several plants and natural products have shown positive effects in alcohol detoxification in vivo studies and clinical trials. These increase the ability of ADH and ALDH in the liver and reduces the blood alcohol level [14]. After drinking, alcohol is primarily metabolized in the liver after being absorbed from the stomach. Under normal conditions, alcohol is mainly metabolized via the ADH pathway [52]. Thus, we measured the activities of ADH and ALDH to confirm the biochemical enzymatic reactions that decompose alcohols and acetaldehyde. ADH and ALDH activities in the blood were significantly further increased in the HY_IPA group after alcohol intake, indicating that HY_IPA suppressed alcohol and acetaldehyde accumulation. ADH oxidizes ethanol to form acetaldehyde, which lowers blood ethanol concentration [53]. However, if only ADH increased and ALDH did not, the concentration of acetaldehyde increased. Thus, acetaldehyde is more reactive and toxic than alcohol and is a greater cause of hangovers. Therefore, ADH and ALDH levels must be increased to eliminate hangover symptoms effectively [54]. 

Among the free amino acids in ice plants, GABA is a non-protein amino acid and neurotransmitter in the brain and spinal cord. Animal experiments have demonstrated that the GABA-rich Smilax extract (FSC) improves alcohol metabolism and reduces alcohol-induced liver damage. FSC improved hangovers by stimulating hepatic alcohol metabolism, commercially acclaimed for its anti-alcoholism effects [55]. In traditional Chinese medicine, *P. lobata* flowers (*Puerariae Flos*) have been used primarily to relive alcohol related problems and injury of liver [56,57]. *P. lobata* flowers contain various isoflavones, including kakkalide, tectoridin, and tectorigenin [58]. The isoflavonoid fractions isolated from *Puerariae Flos* suppresses the increase in blood alcohol, acetaldehyde, and ketone concentrations induced by alcohol intake [59,60]. Moreover, it has been used to improve hangover symptoms traditionally associated with alcohol consumption [61]. ADH and ALDH activities in the liver are further enhanced by pretreatment with the extract after alcohol administration. Therefore, alcohol clearance could be accelerated by FPE pretreatment, and coincides with the highest blood alcohol concentration suppressed, and the prolonged resistance time and shortened poisoning time in mice after EtOH administration. These results demonstrated that FPE accelerates alcohol metabolism by upregulating the ability of alcohol related metabolizing enzymes [62]. Tectoridin, an isoflavone glycoside isolated from the flowers of *Pueraria lobata*, showed the effect of protecting the liver diseases such as alcoholic hepatic steatosis by a significantly reducing ALT, AST, and triglyceride levels in serum, modulating the disturbance of peroxisome proliferators activated receptor α pathway and alleviating hepatic mitochondria disorder in mice [63].

In an experiment measuring the changes in alcohol concentration, ADH, and ALDH in HepG2 cells, the *Artemisia capillaris*-treated group showed a significantly higher alcohol decomposition effect than the alcohol-treated group [21]. In addition, *Artemisia capillaris* is an effective substance for liver disease owing to its high antioxidant activity; in particular, liver protective effects have been reported by in vitro experiments [64]. The *Artemisia* mugwort genus is widely used in food and medicine and contains physiologically active flavonoids such as scoparone, capilartemisin A and B, cirsimaritin, genkwanin, and rhamnocitrin. *Artemisia* has antioxidant, anti-inflammatory, analgesic, antibacterial, and lipid peroxidative effects. Mugwort extract increased antioxidant enzymes activity, such as sodium dismutase, catalase, and glutathione S-transferase [65]. Moreover, mugwort has been used to cure hangovers and improve liver function because it contains catechins, which are phenolic components [66]. Therefore, in this study, GABA, flavonoids and several physiologically active substances present in plant-based extract mixture act synergistically to promote ADH and ALDH activity, and thereby HY_IPA can effectively relieve hangover symptoms by reducing alcohol and acetaldehyde concentrations in the blood. Based on additional experimental evidence, future directions of research should be conducted to confirm the effectiveness of HY_IPA at various doses and further studies is still needed on the relevance of the hangover symptom relief effect by discovering the functional substances of HY_IPA and identifying the mechanism of action.

## 5. Conclusions

The results provide clinical evidence to support the hangover relief effect of HY_IPA. A significant difference between the intake groups in the first and second efficacy evaluation variables was observed, indicating that HY_IPA improves hangover symptoms. No severe adverse reactions occurred. Therefore, HY_IPA can be safely used as a potential anti-hangover ingredient that reduces blood alcohol, acetaldehyde levels, and other hangover symptoms by improving ADH and ALDH enzymatic activities. 

## Figures and Tables

**Table 1:** Baseline characteristics of study participants.

		HY_IPA N = 37	Placebo N = 39	Total N = 76	*p*-Value
Sex n (%)	Male Female	23 (62.16) 14 (37.84)	22 (56.41) 17 (43.59)	45 (59.21) 31 (40.79)	0.6101 (C)
Fertility n (%)	Yes No	14 (100.00) 0 (0.00)	17 (100.00) 0 (0.00)	31 (100.00) 0 (0.00)	
Age (Years)	Mean ± SD Min, Max	29.00 ± 5.70 21.00, 40.00	28.69 ± 4.59 20.00, 39.00	28.84 ± 5.13 20.00, 40.00	0.7958 (T)
Exercise n (%)	No 1~2 per week 3~4 per week 5~6 per week Every day	7 (18.92) 12 (32.43) 17 (45.95) 1 (2.70) 0 (0.00)	10 (25.64) 15 (38.46) 13 (33.33) 0 (0.00) 1 (2.56)	17 (22.37) 27 (35.53) 30 (39.47) 1 (1.32) 1 (1.32)	0.5576 (F)
Smoking n (%)	No Over 1 year Less than 1 year Smoking	18 (48.65) 2 (5.41) 3 (8.11) 14 (37.84)	22 (56.41) 3 (7.69) 1 (2.56) 13 (33.33)	40 (52.63) 5 (6.58) 4 (5.26) 27 (35.53)	0.6844 (F)
Quantity of Alcohol consumption (g/week)	Mean ± SD Min, Max	68.42 ± 34.08 22.61, 144.13	69.59 ± 43.29 8.83, 192.17	69.02 ± 38.81 8.83, 192.17	0.9386 (W)
Frequency of Alcohol consumption (times/week)	Mean ± SD Min, Max	1.54 ± 0.69 1, 4	1.62 ± 0.75 1, 4	1.58 ± 0.72 1, 4	0.6958 (W)
Height (cm)	Mean ± SD Min, Max	170.65 ± 8.89 151.80, 188.00	169.06 ± 8.85 153.30, 183.10	169.83 ± 8.85 151.80, 188.00	0.4349 (T)
BMI (kg/m^2^)	Mean ± SD Min, Max	22.66 ± 2.01 19.10, 24.90	22.50 ± 1.76 18.90, 24.70	22.58 ± 1.88 18.90, 24.90	0.3968 (W)

Values are presented as mean ± standard deviation or number (%). (T) *p*-value for two-sample *t*-test, (W) *p*-value for Wilcoxon rank sum test, (C) *p*-value for chi-square test, and (F) *p*-value for Fisher’s exact test.

**Table 2:** Biochemical measurements.

Variable	HY_IPA (N = 37)			Placebo (N = 39)			*p*-Value
	Baseline	Visit 2 (Day 1)	Change	Baseline	Visit 2 (Day 1)	Change	
Systolic pressure (mmHg)	122.03 ± 11.12	110.57 ± 10.82	−11.27 ± 13.18	123.10 ± 10.90	110.92 ± 9.96	−12.03 ± 9.60	0.7752 (T)
Diastolic pressure (mmHg)	74.59 ± 7.04	70.41 ± 7.76	−3.76 ± 8.72	74.40 ± 7.93	69.67 ± 7.25	−4.69 ± 8.43	0.6358 (T)
Pulse (bmp)	79.05 ± 10.06	74.41 ± 10.21	−4.59 ± 12.02	79.93 ± 10.69	75.79 ± 9.14	−3.87 ± 10.30	0.7788 (T)
Weight (kg)	66.01 ± 11.27	66.19 ± 11.98	−0.37 ± 1.28	64.65 ± 9.84	64.15 ± 10.19	−0.56 ± 1.09	0.6361 (W)
AST (GOT) (U/L)	20.82 ± 5.54	21.84 ± 7.10	0.84 ± 6.06	21.98 ± 6.07	22.10 ± 5.96	−0.03 ± 6.01	0.5139 (W)
ALT (GPT) (U/L)	15.44 ± 8.49	17.57 ± 8.39	1.73 ± 3.65	18.00 ± 7.93	18.21 ± 7.55	0.28 ± 6.75	0.0323 (W)
γ-GPT (U/L)	23.44 ± 12.15	22.11 ± 12.12	−1.76 ± 3.88	24.93 ± 12.89	23.64 ± 12.54	−1.46 ± 3.87	0.3194 (W)
Total cholesterol (mg/dL)	193.13 ± 24.33	182.54 ± 25.92	−11.51 ± 15.74	193.13 ± 29.88	187.74 ± 32.08	−5.72 ± 18.07	0.1411 (T)
Glucose (mg/dL)	91.08 ± 4.57	89.16 ± 5.54	−1.89 ± 7.28	90.03 ± 6.20	88.56 ± 7.42	−1.49 ± 7.67	0.8143 (T)
Total protein (g/dL)	7.33 ± 0.29	6.92 ± 0.36	−0.41 ± 0.35	7.29 ± 0.30	6.98 ± 0.29	−0.32 ± 0.33	0.2488 (T)
BUN (mg/dL)	12.34 ± 2.92	13.11 ± 1.57	0.61 ± 3.12	11.58 ± 3.28	12.68 ± 2.86	1.08 ± 2.66	0.4831 (T)
Creatinine (mg/dL)	0.79 ± 0.19	0.76 ± 0.15	−0.03 ± 0.10	0.77 ± 0.20	0.76 ± 0.17	−0.01 ± 0.09	0.3623 (T)
Uric acid (mg/dL)	5.38 ± 1.22	5.50 ± 1.13	0.11 ± 0.61	5.36 ± 1.23	5.45 ± 1.14	0.07 ± 0.56	0.7440 (T)
Ca (mg/dL)	9.23 ± 0.27	8.95 ± 0.29	−0.29 ± 0.30	9.19 ± 0.30	9.00 ± 0.28	−0.20 ± 0.29	0.1564 (T)

Comparison between groups: *p*-value for the two-sample *t*-test (T) or Wilcoxon rank-sum test (W).

**Table 3:** Mean Acute Hangover scale and individual hangover symptom scores.

	HY_IPA N = 37 Mean ± SD	Placebo N = 39 Mean ± SD	*p*-Value
Total score	5.24 ± 5.78	18.54 ± 18.50	<0.0001 (W)
Hangover	0.57 ± 0.96	2.41 ± 2.45	0.0002 (W)
Thirst	1.86 ± 2.00	4.26 ± 2.22	<0.0001 (W)
Tired	1.32 ± 1.51	3.56 ± 2.19	<0.0001 (W)
Headache	0.65 ± 1.18	1.90 ± 2.50	0.0177 (W)
Dizziness/faintness	0.11 ± 0.46	1.05 ± 2.01	0.0043 (W)
Loss of appetite	0.35 ± 0.89	1.56 ± 2.39	0.0106 (W)
Stomachache	1.56 ± 2.39	1.36 ± 2.35	0.0007 (W)
Nausea	0.16 ± 0.69	1.23 ± 2.30	0.0032 (W)
Heart racing	0.08 ± 0.36	1.21 ± 2.41	0.0074 (W)

Comparison between groups: *p*-value for the Wilcoxon rank-sum test (W).

**Table 4:** Comparison of Acute Hangover Scale between males and females.

Indicators	Male (N = 45)			Female (N = 31)		
	HY_IPA (N = 23)	Placebo (N = 22)	*p*-Value	HY_IPA (N = 14)	Placebo (N = 17)	*p*-Value
Total score	5.30 ± 6.64	17.32 ± 19.26	0.0102 (W)	5.07 ± 4.18	20.06 ± 17.95	<0.001 (W)
Hangover	0.52 ± 1.04	1.73 ± 2.27	0.0306 (W)	0.64 ± 0.84	3.29 ± 2.44	<0.001 (W)
Thirst	2.13 ± 2.34	4.18 ± 2.36	0.0055 (W)	1.43 ± 1.22	4.35 ± 2.09	<0.001 (W)
Tired	1.22 ± 1.68	3.09 ± 2.33	0.0038 (W)	1.5 ± 1.22	4.18 ± 1.88	<0.001 (W)
Headache	0.48 ± 0.99	1.59 ± 2.56	0.0671 (W)	0.86 ± 1.46	2.29 ± 2.44	0.053 (W)
Dizziness/faintness	0.09 ± 0.42	1.18 ± 2.28	0.0372 (W)	0.14 ± 0.53	0.88 ± 1.65	0.0979 (W)
Loos of appetite	0.48 ± 1.04	1.73 ± 2.37	0.0311 (W)	0.14 ± 0.53	1.35 ± 2.47	0.0656 (W)
Stomachache	0.13 ± 0.63	1.41 ± 2.20	0.0146 (W)	0.14 ± 0.53	1.24 ± 2.61	0.1100 (W)
Nausea	0.22 ± 0.85	1.18 ± 2.44	0.0914 (W)	0.07 ± 0.27	1.29 ± 2.17	0.0348 (W)
Heart racing	0.04 ± 0.21	1.23 ± 2.39	0.0306 (W)	0.14 ± 0.53	1.18 ± 2.51	0.1154 (W)

Comparison between groups: *p*-value for the Wilcoxon rank-sum test (W).

**Table 5:** Variation in blood alcohol and acetaldehyde concentration between the HY_IPA and placebo groups.

		HY_IPA N = 37 Mean ± SD	Placebo N = 39 Mean ± SD	*p*-Value
	AUC	0.7977 ± 0.2297	0.8011 ± 0.2049	0.9454 (T)
Alcohol level (%)	T_max_	1.3649 ± 0.8283	0.9872 ± 0.6588	0.0497 (W)
	C_max_	0.1496 ± 0.0346	0.1805 ± 0.0635	0.0586 (W)
	AUC	0.0094 ± 0.0028	0.0116 ± 0.0041	0.0283 (W)
Acetaldehyde level (%)	T_max_	0.9122 ± 1.2194	1.0577 ± 2.1116	0.5494 (W)
	C_max_	0.0015 ± 0.0005	0.0017 ± 0.0006	0.0180 (W)

Comparison between groups: *p*-value for the two-sample *t*-test (T) or Wilcoxon rank-sum test (W). AUC, area under the curve; C_max_, peak alcohol blood concentration; T_max_, time to reach the peak blood concentration.

**Table 6:** Effects of HY_IPA supplementation on blood ADH activities in participants.

		Total N = 76 Mean ± SD	HY_IPA N = 37 Mean ± SD	Placebo N = 39 Mean ± SD	*p*-Value
	0 h	2.5 ± 0.77	2.97 ± 0.59	2.11 ± 0.64	<0.0001
	1 h	3.03 ± 1.13	3.96 ± 0.81	2.24 ± 0.53	<0.0001
ADH (mU/mL)	2 h	2.65 ± 0.67	2.91 ± 0.74	2.47 ± 0.40	0.0012
	4 h	2.23 ± 0.59	2.23 ± 0.66	2.27 ± 0.46	0.9755
	6 h	2.04 ± 0.59	1.88 ± 0.57	2.23 ± 0.53	0.0199

Compared between groups; *p*-value for two sample *t*-test.

**Table 7:** Effects of HY_IPA supplementation on blood ALDH activities in participants.

		Total N = 76 Mean ± SD	HY_IPA N = 37 Mean ± SD	Placebo N = 39 Mean ± SD	*p*-Value
	0 h	28.82 ± 0.6.4	32.15 ± 5.47	25.67 ± 5.62	<0.0001
	1 h	35.1 ± 9.88	42.65 ± 6.84	27.95 ± 6.35	<0.0001
ALDH (mU/mL)	2 h	32.44 ± 7.14	35.30 ± 6.54	29.72 ± 6.68	<0.0001
	4 h	30.33 ± 6.66	29.86 ± 6.32	30.78 ± 7.03	0.5484
	6 h	24.14 ± 6.7	22.40 ± 6.36	25.79 ± 6.67	0.026

Compared between groups; *p*-value for two sample *t*-test.

**Table 8:** Statistically correlation between 1-item of Hangover score and concentration of alcohol, acetaldehyde, and ADH, ALDH activity.

Subject	Alcohol	Acetaldehyde	ADH	ALDH
HY_IPA	0.3570 *p =* 0.0301 *	0.4335 *p =* 0.0074 **	−0.1529 *p =* 0.3662	0.489 *p =* 0.0021 **
Placebo	0.4629 *p =* 0.0030 **	0.0233 *p =* 0.8879	−0.0093 *p =* 0.9554	−0.0015 *p =* 0.9928

Significant correlations are indicated by * (*p* < 0.05) or ** (*p* < 0.01).

## Data Availability

The data presented in this study are available.
